# Emotional well-being in Charles Bonnet syndrome: exploring associations with negative affect, loneliness and quality of life

**DOI:** 10.1177/25158414241275444

**Published:** 2024-09-26

**Authors:** Bethany Higgins, Deanna Taylor, David Crabb, Tamsin Callaghan

**Affiliations:** Optometry and Visual Sciences, School of Health & Psychological Sciences, City, University of London, London, UK; Optometry and Visual Sciences, School of Health & Psychological Sciences, City, University of London, London, UK; Optometry and Visual Sciences, School of Health & Psychological Sciences, City, University of London, London, UK; NIHR Royal Free Clinical Research Facility, Research and Development, Royal Free London NHS Foundation Trust, 02/62, Second Floor, Clinic Block, Royal Free Hospital, Pond Street, London NW3 2QG, UK; Optometry and Visual Sciences, School of Health & Psychological Sciences, City, University of London, London, UK

**Keywords:** Charles Bonnet syndrome, loneliness, negative affect, quality of life, well-being

## Abstract

**Background:**

Charles Bonnet syndrome (CBS) is a condition characterised by the occurrence of vivid and complex visual hallucinations in individuals with visual impairment.

**Objective:**

To explore the relationship between emotional distress and the perceived impact of CBS symptoms on participants’ lives. We tested the hypothesis that heightened negative affect was associated with a more negative appraisal of CBS symptoms, increased self-reported loneliness, and poorer quality of life (QOL).

**Design:**

Cross-sectional.

**Methods:**

Participants recruited predominantly via vision-related charities rated their hallucinations and their impact on a Likert scale. Loneliness and negative affect were assessed with the Three-Item Loneliness Scale and Positive and Negative Affect Schedule. Health index (EQ-5D-3L) and vision-related QOL (VF-9) were also assessed. Correlation analysis and multi-variable regression determined the relation between survey responses.

**Results:**

The majority of 126 respondents (81%) were aged 65+ years and 84% reported active CBS symptoms. Fifty-five percent of respondents rated impact of CBS as negative and no-one rated the impact as ‘very pleasant’. A statistically significant correlation was found between impact of CBS and negative affect (*p* ⩽ 0.001; rho = −0.34) and impact of CBS and loneliness (*p* = 0.017; rho = −0.21). The relation between negative affect and CBS impact remained statistically significant when accounting for the impact of loneliness and the relationship between loneliness and CBS effect (*p* = 0.002, adj *R*^2^ = 0.1). A statistically significant correlation between loneliness and negative affect (*p* ⩽ 0.001; rho = 0.55) was also found.

**Conclusion:**

Respondents experiencing negative emotions were more likely to perceive the impact of CBS symptoms as negative and report greater feelings of loneliness. Negative affect is an important consideration when assessing people with CBS.

## Plain language summary

Understanding the impact of visual hallucinations in Charles Bonnet syndrome

Charles Bonnet Syndrome (CBS) is a condition where people with vision problems experience vivid and complex visual hallucinations. In this study, we wanted to see how feeling upset or lonely might affect how people with CBS view their symptoms. We asked 126 adults, most of whom were over 65 years old, about their hallucinations and how they felt about them. We also asked about feelings of loneliness and general emotional well-being. We found that the more negative emotions people felt, the more they tended to see their CBS symptoms in a negative light and feel lonelier. This suggests that understanding and addressing negative emotions is crucial when helping people with CBS.

## Introduction

Charles Bonnet syndrome (CBS) refers to visual hallucinations experienced by people with a visual impairment. Since the 1990s, CBS has referred only to complex hallucinations such as people or animals. However, recently its usage has expanded to include other types of hallucinations such as simple dots or flashes. For the purposes of our study, we adopted this broader definition, including both complex and simple visual hallucinations. It has been estimated that as many as 20% of people with sight loss experience CBS^1^ and old age is considered a risk factor.^2^ People with CBS have intact cognition, do not have a psychological impairment and are aware that the visual hallucinations are not real. Yet, visual hallucinations are habitually associated with cognitive decline and it has been suggested the stigma surrounding them results in hesitancy to disclose hallucinations to family, friends and medical professionals.^3^ Social isolation has been suggested to influence hallucination manifestation.^4^ While some people do not find the visual hallucinations problematic, it has been reported that a third of people with CBS find it has a negative impact on their life, coined ‘negative-outcome’ CBS by Cox and ffytche.^5^

Visual impairment has health implications that extend beyond the functioning of the eyes and is linked to a decrease in functional capabilities, a greater risk of falls, loneliness, and mortality.^6^ Increased rates of depression and feelings of negative affect have been reported in people with a visual impairment from large population studies.^7–9^ Over the next three decades, there is projected to be a significant increase in global rates of moderate-to-severe vision impairment, which is expected to double in prevalence.^10^ As the rates of visual impairment surge, so will CBS. Consequently, it is imperative that our understanding of the impact of CBS on quality of life (QOL) is broadened so that suitable support systems can be put in place.

Quality of life is a complex concept and can be defined as a subjective metric of someone’s own perceived well-being. It is influenced by health and disability,^11^ relationships^12^ and a person’s unique situation and expectations. Patient-reported outcome measures (PROMs) are increasingly recognised as a method to meaningfully capture a person’s subjective view of their own health or QOL with a standardised approach.^13^ As a result, the use of PROMs has increased in medical research and their use as an endpoint in clinical trials has grown.^14^ It is well established that presence of a visual impairment is associated with poorer QOL^15–17^ that has been found to worsen with impairment progression.^18^ The added experience of CBS has been found to be a significant predictor of poorer QOL and emotional distress in people with a visual impairment, even when visual functionality is controlled for.^19^

Greater levels of loneliness have been documented among older adults^20^ and more so in visually impaired cohorts.^21^ Loneliness reduces QOL^20^ and for those with CBS, exacerbates hallucinations.^4^ It has been suggested that sensory deprivation due to being alone may be partially responsible for visual hallucinations.^3^ For example, an increase in social isolation during the COVID-19 was associated with an increase in hallucinatory episodes.^4^

As assessments of QOL are coloured by people’s expectations and are reliant on self-report, it follows that aversive moods states such as dysphoria, anger or contempt (i.e. negative affect) or cheerfulness and happiness (positive affect) would impact how one would perceive and ultimately report on their health and overall well-being.^22,23^ Or indeed, how someone with CBS would perceive the way visual hallucinations impact their life. In fact, elevated levels of negative affect are believed to exert an impact on health by imposing physical strain on the body and by amplifying our propensity to make unhealthy choices.^24,25^ Individuals with higher levels of negative affect have found to report poorer self-perceived health and greater symptoms.^22^ For example, increased negative affect has been linked to poorer QOL and metabolic control in people with Type 2 diabetes.^26^ Yet, it has not been explored empirically if mood of an individual relates to how they perceive the impact of their CBS symptoms on QOL.

The primary aim of this study is to assess the relation between self-reported impact of CBS, levels of positive and negative affect and feelings of loneliness. To achieve this, we utilise standardised questionnaires: The Positive and Negative Affect Schedule (PANAS)^27^ and the Three-Item Loneliness Scale.^28^ The secondary aim is to evaluate additional measures of QOL, including overall health-related QOL using the EuroQuol5D (EQ-5D-3L), health index^29^ and vision-related QOL (Visual Function Questionnaire (VF-9)^30^). We test the hypothesis that greater levels of negative affect correlate with a greater likelihood to report the impact of CBS symptoms as negative and greater levels of loneliness, as well as poorer self-reported QOL.

## Methods

### Participant recruitment

Recruitment was conducted via social media adverts and through the help of national and local vision-related charities including the Macular Society, Esme’s Umbrella, RNIB and Lincoln and Lindsey Blind Society (see Acknowledgements). Participants who reported having a visual impairment were invited to take part. A structured survey was accessible from May 2022 to April 2023 and was available both online using Qualtrics software (Qualtrics, Provo, UT, USA) and in large text paper format. For people unable to access the questionnaire, a member of the research team (BH) administered it over the phone. To be included in analysis, participants were required to be aged 18 years old or over, have impaired vision (of any cause), self-reported CBS (either past symptoms or ongoing) and no self-reported cognitive impairments. No formal diagnosis of CBS by a clinician was necessary for inclusion and any form of visual hallucination (both simple and complex) was accepted. A target sample size of 100 participants in total was set to detect a standardised difference between any pair of groups of 0.6 (a medium-to-large effect size) with 80% power at a 5% (two tailed) significance level. Power calculations were made using the pwr package in the open-source statistical environment R (version 3.5.3).

### Survey development

**Figure 1.**
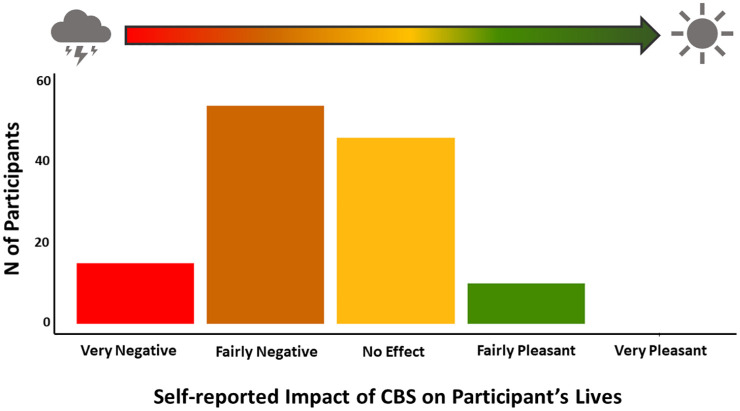
Bar plot of frequency of responses on the impact of CBS on participants’ lives. No-one rated impact of CBS as ‘very pleasant’. CBS, Charles Bonnet syndrome.

A novel survey to examine CBS characteristics, effect of CBS on QOL and mood was designed based on the existing literature and featured all questions previously developed by Cox and ffytche.^5^ The survey included demographic and clinical characteristic information, characteristics of and attitudes towards hallucinations and, importantly, the reported impact that CBS has on participants’ life, rated on a 5-level Likert scale from very negative to very pleasant (see Figure 1). Overall health-related QOL was assessed using EQ-5D-3L health index, a continuous index typically ranging from −0.59 to 1.0 in the United Kingdom, with 1.0 representing full health and negative values representing health states considered worse than death.^29^ Vision-related QOL was assessed via the abbreviated Visual Function Questionnaire (VF-9; with 0 being very good vision-related QOL and 100 being very poor vision-related QOL).^30^ Levels of positive and negative affect were assessed using The Positive and Negative Affect Schedule (PANAS; with scores ranging from 10 to 50, with lower scores representing lower levels of positive or negative affect)^27^ and self-reported loneliness was analysed using the Three-Item Loneliness Scale (with 0 being not lonely at all and 9 being very lonely).^28^ Participant information was anonymised before being entered into a secure computer database. The full survey used is available in supplemental materials.

### Patient and public involvement

Patient involvement was central to the design of this study. A focus group was held with two visually impaired people with age-related macular degeneration who offered feedback on the survey design and accessibility. Their feedback included advice such as removing the lines for people to write their answers on as this was complicated by metamorphopsia. Instead, we were encouraged to replace these with boxes for people to fill their answers in. The questionnaire design was amended in light of this feedback and was sent out again to focus group members for approval. This final version was approved and adopted into the study. To ensure accessibility of the online version of the questionnaire, the charities Macular Society and Bravo Victor supported the study by checking the survey was screen-reader friendly, using popular types of screen-readers including NonVisual Desktop Access (Assistivlabs, Delaware, USA) and Job Access with Speech (Freedom Scientific, Florida, USA). Feedback was that the questionnaire was readable using these technologies. To ensure the survey would be accessible to everyone including those without access to a computer nor comfortable filling it in on paper, a member of the research team (BH) was available to administer the survey over the phone.

### Statistical analysis

All data analyses were performed in R version 4.2.2 (http://www.r-project.org/) under R Studio (RStudio, Boston, MA, USA) including the use of the ggplot2 package. Spearman correlation analysis and multiple variable regression were conducted to assess the relationships between survey responses. Ninety-five percent proportional confidence intervals (CIs) are given using the Wald method. The median negative affect score from normative data collected by Crawford and Henry from 1003 members of the general public (*n* = 466 men, *n* = 537 women) with a mean age of 43 years (range 18–91 years) was 14 and is used as a guide of how the general public would score.^31^ The main analysis was conducted on the whole cohort (people with active CBS as well as those who report hallucinations have now stopped). We also conducted a separate analysis on people who report CBS as ‘active’ and people with both active hallucinations who report having negative-outcome CBS.

## Results

This survey includes 126 adults from the UK with CBS, 81% of respondents were aged over 65 years (97 females, 28 males). See Table 1 for demographic and clinical details.

**Table 1.** Self-reported demographic and clinical details of *n* = 126 participants.

Demographic and clinical details	Frequency (%)
Age (years)	
18–35	4 (3%)
36–50	4 (3%)
51–65	12 (10%)
Over 65	106 (84%)
Gender	
Male	28 (22%)
Female	97 (77%)
Unknown	1 (1%)
Sex (assigned at birth)	
Male	28 (22%)
Female	97 (77%)
Unknown	1 (1%)
Visual impairment	
Age-related macular degeneration	72 (57%)
Glaucoma	13 (10%)
Diabetic retinopathy	6 (5%)
Brain/eye tumour	4 (3%)
Other	28 (22%)
Unknown	10 (8%)
Binocular impairment	
Binocular	106 (84%)
Monocular	20 (16%)

**Figure 2.**
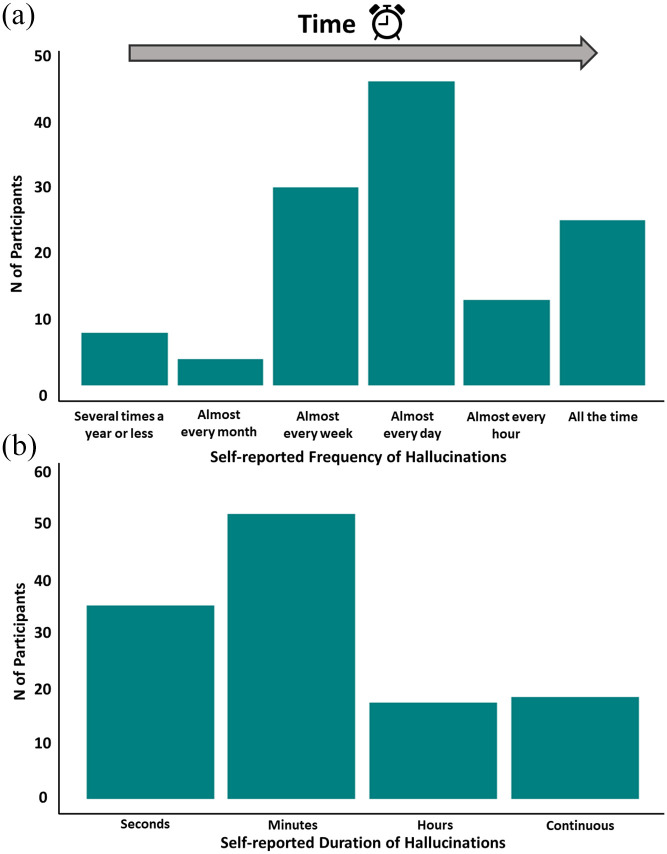
Bar plot of reported hallucination frequency (a) and duration (b).

When participants from the whole cohort were asked to rate the impact that CBS has on their life, over half of the respondents (55%, n70 (95% CI 46%, 64%)) rated CBS impact as negative. These n70 participants can be described as experiencing negative-outcome CBS.^5^ No participants rated the CBS impact as ‘very pleasant’ on their lives (Figure 1). Eighty-nine percent (*n* = 113, (95% CI 83%, 94%)) of the n126 participants reported their CBS to be active while the remaining 10% of participants (*n* = 13, (95% CI 6%, 17%)) reported their hallucinations to have stopped. Seventy-five percent of participants (*n* = 95, (95% CI 67%, 82%)) reported experiencing complex hallucinations such as faces, animals, figures or objects on more than one occasion. In terms of hallucination frequency and length, n46 participants reported experiencing a hallucination every day and n53 participants reported hallucination durations of minutes (Figure 2). A statistically significant negative correlation was identified between CBS impact and hallucination frequency (*p* = 0.024; rho = −0.20), but a statistically significant correlation was not identified between CBS impact and length of hallucination (*p* = 0.077; rho = −0.16).

### Primary analysis: Impact of CBS, negative affect and loneliness

**Figure 3.**
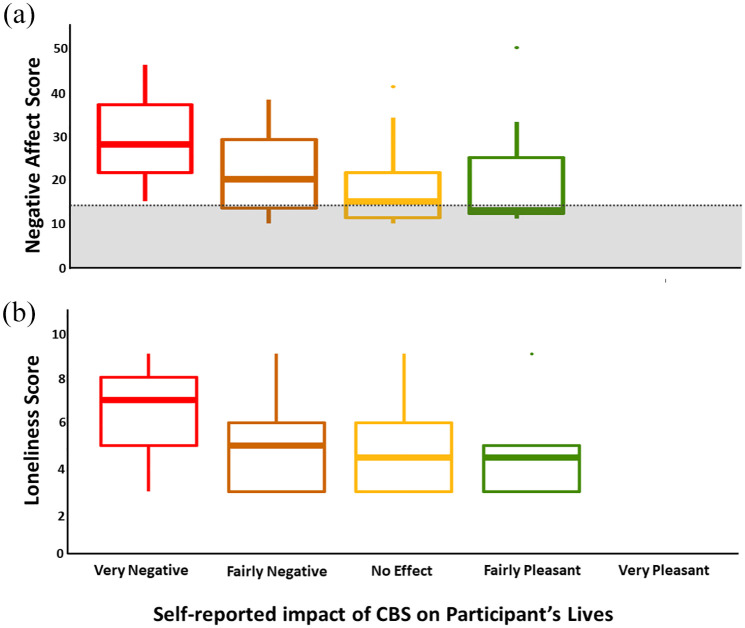
Boxplots comparing the relationship between impact of CBS on participants’ lives with negative affect score (a) and loneliness score (b). The median Negative Affect score for n1003 members of the general public was reported as 14 (Crawford and Henry^31^). This score is shown by the grey dotted line on the plot. All participants that rated impact of CBS as ‘very negative’ scored a higher level of negative affect than the mean score of the general public. CBS, Charles Bonnet syndrome.

A statistically significant moderate negative correlation was identified between CBS impact and negative affect (*p* < 0.001; rho = −0.34) and participants who rated CBS impact as ‘very negative’ were all found to self-report a higher level of negative affect compared to the general public (see Figure 3).^31^ A statistically significant weak negative correlation was also identified between CBS impact and loneliness (*p* = 0.017; rho = −0.21). A statistically significant moderate positive correlation between loneliness and negative affect (*p* < 0.001; rho = 0.55) was also found. Multiple variable regression revealed the relationship between negative affect and CBS impact remains statistically significant even when accounting for impact of loneliness and the relationship between loneliness and CBS impact (*p* = 0.002, adj *R*^2^ = 0.1).

### Secondary analysis: Measures of quality of life

A statistically significant correlation was found between both positive affect and negative affect, between positive affect and loneliness and between positive affect and health index (all *p* < 0.01), but not between positive affect and CBS impact nor vision-related QOL. A statistically significant correlation was identified between vision-related QOL and negative affect, loneliness and health index and CBS impact (all *p* < 0.01). A statistically significant correlation was also found between health index and negative affect, loneliness and CBS impact (all *p* < 0.01). See Table 2 for full details of correlational analyses.

**Table 2.** Correlational analysis of outcomes from questionnaire.

	CBS impact	Loneliness	Negative affect	Positive affect	Vision-related QOL
Loneliness	***p* = 0.017** **rho = −0.21**	–	–	–	–
Negative affect	***p* < 0.001** **rho = −0.34**	***p* < 0.001** **rho = 0.55**	–	–	–
Positive affect	*p* = 0.14 rho = −0.34	***p* < 0.01** **rho = −0.30**	***p* < 0.001** **rho = −0.30**	–	–
Vision-related QOL	***p* < 0.001** **rho = 0.34**	***p* < 0.001** **rho = −0.39**	***p* < 0.01** **rho = −0.29**	*p* = 0.1 rho = 0.15	–
EQ-5D	***p* < 0.01** **rho = 0.25**	***p* < 0.001** **rho = 0.41**	***p* < 0.001** **rho = −0.34**	***p* < 0.001** **rho = −0.36**	***p* < 0.001** **rho = 0.45**

CBS, Charles Bonnet syndrome; EQ-5D, EuroQuol5D; QOL, quality of life. *p* values in bold indicate a statistical significant correlation.

### Subgroup analysis

When analysis was conducted on data from participants with active CBS only (89%, n113), a statistically significant correlation remained between CBS impact and loneliness (*p* = 0.013; rho = −0.23) and negative affect (*p* < 0.001; rho = −0.34). A statistically significant positive correlation also remained between loneliness and negative affect (*p* < 0.001; rho = 0.51). Furthermore, analysis conducted on data from participants with active negative-outcome CBS only (55%, n70) found a marginally less statistically significant negative correlation remained between CBS impact and negative affect (*p* < 0.01; rho = −0.32), but a more statistically significant correlation between CBS impact and loneliness (*p* = 0.004; rho = −0.24). There was no change in correlation between loneliness and negative affect (*p* < 0.001; rho = 0.51). See Supplemental Figures 1 and 2 for all comparisons.

## Discussion

To the author’s knowledge, this study is the first original report assessing the relationship between self-reported impact of visual hallucinations and emotional well-being, loneliness and parameters of QOL. In this large (*n* = 126) cohort, over half of the respondents rated the impact of CBS on their life as negative and no-one reported the hallucinations as having a ‘very pleasant’ impact.

Participants who reported CBS impact as negative were more likely to report higher levels of negative affect, while the opposite was true for participants who reported low levels of negative affect (e.g. a state of calmness and tranquillity^27^). Furthermore, participants who reported the impact of CBS as ‘very negative’ were found to have a negative affect score higher (therefore worse) than the mean of the general population.^31^

Individuals who exhibit higher levels of negative affect are more inclined to experience negative emotions.^27^ These include an increased propensity for worry, feelings of anger and frustration, and sadness. The emotions can be short-term or persistent, like depression that can endure over an extended period.^32^ Additionally, people with higher levels of negative affect may be more likely to perceive self-criticism and feel less resistant to stress.^33^ There is evidence supporting the association between higher levels of negative affect and poorer self-perceived health, as well as increased symptomatology.^22,26^ The classic Symptom-Perception Hypothesis^34^ is widely accepted as the primary explanation for the association between negative affect and symptoms, which suggests that individuals with heightened levels of negative affect tend to be focussed internally. Therefore, this amplifies their sensitivity to minor somatic sensations and explains the inflation of symptoms reporting. Prior research has shown that people with CBS report significantly more depression and feelings of anxiety compared to patients without CBS.^35,36^ Yet, our findings are the first to evidence an association between negative affect and perceived negative impact of CBS. This data highlights the need for interventions and support systems to address the negative effects associated with this condition.

Loneliness is a leading health concern, especially in elderly populations. In the visually impaired community, the rate of loneliness is higher. Brunes et al. has reported one-sixth of visually impaired adults experience moderate-to-severe levels of loneliness.^37^ For people with CBS, loneliness can have an aggravating effect on hallucination symptomology. Jones et al. examined the impact of the COVID-19 pandemic and the resulting social isolation on the experiences of 45 people with CBS. The authors found approximately half of the respondents reported an increase in visual hallucinations during the COVID-19 pandemic, which may be attributed to increased loneliness. For example, some participants reported that lilliputian hallucinations (miniature-sized hallucinations) had grown in magnitude and became increasingly challenging to disregard.^4^ Notably, to measure loneliness the authors utilised a singular self-labelling item on the survey. While this is typical of epidemiological studies, a standardised loneliness questionnaire would be more robust. In our study, the Three-Item Loneliness Scale was used to quantify levels of loneliness, developed from the Revised UCLA Loneliness Scale.^28^ Respondents who identified as socially isolated also reported experiencing more negative mood states and felt the impact of their CBS was negative on their life, compared to those who did not report high levels of social isolation. These results suggest that social isolation is linked with the negative emotional experiences associated with CBS and hence adds weight to Jones et al. findings.

Our results revealed the relationship between negative affect and CBS impact remained statistically significant when self-reported loneliness was controlled for. This finding could indicate that loneliness may not play a mediating role in this particular relationship. However, the authors believe that the findings highlight the complex nature of the relationship between negative affect, loneliness, and the impact of CBS. Therefore, addressing the negative emotional consequences of CBS requires considering multiple factors beyond loneliness alone. Considering the role of loneliness and negative affect, management strategies for individuals with negative-outcome CBS should be holistic, focusing on promoting and integrating social connections and reducing feelings of loneliness to improve overall well-being, potentially mitigating the impact of CBS symptoms on emotional well-being.

In a secondary analysis, we repeated our analyses in subgroups of participants who reported active hallucinations (*n* = 113) and those with active negative-outcome CBS (people who were experiencing negative experiences of CBS when completing the survey; *n* = 70). The results from these cohorts continued to demonstrate higher levels of negative affect correlated with a negative experience of CBS symptoms. Yet, the research revealed a stronger, significant correlation between perceived negative impact of CBS and increased levels of loneliness in individuals with active negative-outcome CBS. This suggests that social isolation plays a crucial role in the manifestation and impact of negative-outcome CBS symptoms in this subgroup.

Our study found that not only did a self-reported negative impact of CBS correlate with negative affect and loneliness, but also poorer QOL when assessed by standardised metrics of health-related QOL^29^ and vision-specific QOL.^38^ This finding supports recent data from Randeblad et al. who reported that vision-related QOL (measured via the National Eye Institute Visual Function Questionnaire 25) was significantly lower in glaucoma patients with CBS compared to glaucoma patients without CBS, matched for age and visual function.^39^ Furthermore, Scott et al. reported that people with CBS score poorly on the General Health Questionnaire compared to visually impaired controls, even when VA was largely preserved.^19^

Recognising the correlation between self-reported negative impact of CBS and poorer QOL, healthcare professionals can prioritise interventions aimed at improving QOL in this cohort. This may involve a multidisciplinary approach that addresses both the visual symptoms of CBS and the broader psychosocial impact on individuals’ lives, inclusive of negative affect and loneliness. In addition, the results emphasise the need for healthcare providers to engage in open dialogue with CBS patients, encouraging them to self-report the impact of their condition.

This study’s strengths include a large population sample that enables greater generalisability to the CBS population and increased ability to detect small but meaningful associations. Furthermore, the use of previously validated questionnaires that have undergone rigorous testing to ensure their reliability and validity not only enhances the quality and credibility of this study but allows comparability across existing research. Perhaps most importantly, the Patient and Public Involvement (PPI) used during the study design helped make outcomes relevant and meaningful to the patients and public who are directly affected by the research.

While significant weak-to-moderate correlational relations were identified between loneliness, negative affect and CBS impact, caution should be exercised in assuming a causal relationship from this data. The study demonstrates associations between the variables of interest, but further research is needed to determine the underlying mechanisms driving these associations. Future longitudinal studies are warranted to explore the temporal dynamics of CBS impact, emotional well-being, and loneliness, which this study enables, but does not explore. Participants were screened for cognitive defects based on self-report, possibly impacting the results due to subtle cognitive differences. Additionally, relying on self-reported measures can potentially introduce response bias, however, they allow for direct insight into participants’ perspectives which may not be observable through objective data collection methods. Future research could consider incorporating objective measures, such as physiological markers, to enhance the validity of findings alongside measures of self-report. The cohort was predominantly female. However, in a recent large-scale prevalence study of CBS in people with open-angle glaucoma, gender was not associated with CBS.^40^ Lastly, no ethnicity data was collected in this study, thereby limiting the generalisability of the results. To address this limitation and gain insights into potential ethnic disparities regarding the relationship between negative affect, loneliness and QOL on the impact of CBS, future studies should prioritise working with ethnically diverse populations and the collection of ethnicity data.

In conclusion, respondents who reported experiencing negative moods and/or were reportedly lonelier were more likely to perceive the impact of their CBS symptoms as negative. This highlights the significance of negative affect when evaluating QOL in individuals with CBS. Addressing the emotional well-being and self-perception of individuals affected by CBS are crucial factors in comprehensive assessments of their QOL.

## Supplemental Material

sj-docx-1-oed-10.1177_25158414241275444 – Supplemental material for Emotional well-being in Charles Bonnet syndrome: exploring associations with negative affect, loneliness and quality of life Supplemental material, sj-docx-1-oed-10.1177_25158414241275444 for Emotional well-being in Charles Bonnet syndrome: exploring associations with negative affect, loneliness and quality of life by Bethany Higgins, Deanna Taylor, David Crabb and Tamsin Callaghan in Therapeutic Advances in Ophthalmology

sj-tif-2-oed-10.1177_25158414241275444 – Supplemental material for Emotional well-being in Charles Bonnet syndrome: exploring associations with negative affect, loneliness and quality of life Supplemental material, sj-tif-2-oed-10.1177_25158414241275444 for Emotional well-being in Charles Bonnet syndrome: exploring associations with negative affect, loneliness and quality of life by Bethany Higgins, Deanna Taylor, David Crabb and Tamsin Callaghan in Therapeutic Advances in Ophthalmology

sj-tif-3-oed-10.1177_25158414241275444 – Supplemental material for Emotional well-being in Charles Bonnet syndrome: exploring associations with negative affect, loneliness and quality of life Supplemental material, sj-tif-3-oed-10.1177_25158414241275444 for Emotional well-being in Charles Bonnet syndrome: exploring associations with negative affect, loneliness and quality of life by Bethany Higgins, Deanna Taylor, David Crabb and Tamsin Callaghan in Therapeutic Advances in Ophthalmology

## Declarations

**Ethics approval and consent to participate:** The study was approved on 3rd February 2022 by the City, University of London Optometry Research Ethics Committee (ETH2223-1278) and was conducted according to the tenets of the Declaration of Helsinki. Written, informed consent was obtained from each participant prior to examination. For those who were assessed over the telephone, verbal consent was audio-recorded.

**Consent for publication:** Written, informed consent for publication was obtained from each participant.

**Availability of data and materials:** The questionnaire utilised in this study is available in Supplemental Materials.
