# Supplementary material for: Emotional well-being in Charles Bonnet syndrome: exploring associations with negative affect, loneliness and quality of life
Source: Ther Adv Ophthalmol. 2024 Sep 26;16:25158414241275444. doi: 10.1177/25158414241275444 (PMC11440537; doi:10.1177/25158414241275444)
Supplement: sj-docx-1-oed-10.1177_25158414241275444 – Supplemental material for Emotional well-being in Charles Bonnet syndrome: exploring associations with negative affect, loneliness and quality of life [file sj-docx-1-oed-10.1177_25158414241275444.docx]

**Questionnaire Introduction**

Thank you so much for agreeing to be part of this new research study entitled **‘An investigation into the prevalence and nature of hallucinations in Charles Bonnet Syndrome (CBS)’**

In order for this survey to improve our knowledge about vision problems and how they affect your quality of life, your answers must be as accurate as possible.

There are 6 sections in total. Please take as much time as you need to answer each question. All your answers are confidential. Please circle your answers.

Date questionnaire filled in: _________

**Part A**

**This set of questions is to find out basic information about you and any visual impairment you may have. Please put a ring around your answer.**

1. **How old are you?**

18 – 35 years

36 – 50 years

51 – 65 years

Over 65 years

**2. What is your gender?**

Male

Female

Prefer not to say

Other (please state in the box below)

**3. Is your gender identity the same as the sex you were assigned at birth?**

No

Yes

Prefer not to say

**4. Other than your visual impairment, do you have any other health conditions?**

No

Don’t know

Yes, (please state in the box below)

**5. Do you take any regular medications?**

No

Don’t know

Yes, (please state in the box below)

**6. What is your visual impairment(s) due to?**

Don’t know

I have (please state in the box below)

**7. How long have you had your visual impairment? (Duration in years, months)**

(Please state in the box below)

**8. Does it affect both eyes?**

Both

Left only

Right only

Different types in each eye

**If you do not have macular disease, go straight to Question 10**

**9. If you have macular disease, what type(s) do you have? (Circle all that apply)**

Wet

Dry

Wet & Dry

A juvenile form

Don’t know

I don’t have macular disease

Other (please state in the box below)

**10. Had you heard of Charles Bonnet Syndrome before this questionnaire?**

Yes

No

**11. Do you have Charles Bonnet Syndrome?**

Yes

No

**If you do not have Charles Bonnet Syndrome please go straight to Page 16 and start Part B**

**12. Did you already know about Charles Bonnet syndrome when you first experienced the hallucinations?**

Yes

No

**13. What did you think might be the cause when you first experienced Charles Bonnet Syndrome? (Circle all that apply)**

I had been told it might occur

Thought it must be to do with sight loss

Thought I might have illness such as Alzheimer’s disease

Thought I might have a mental illness.

Did not know what to think

Other (please state in the box below)

**14. If you have consulted a medical professional, what did they say it was? (Circle one that best applies)**

They gave a clear account of Charles Bonnet Syndrome

They were unsure or did not know

I have not consulted a medical professional

They gave a different diagnosis of: (please state in the box below)

Other (please state in the box below)

**15. What difference did finding out about Charles Bonnet Syndrome make to your feelings about the hallucinations? (Circle all that apply)**

Reassured

Relieved

Confused

Angry that I hadn’t been told about it earlier

It made no difference

Other (please state in the box below)

**16. How did you first find out about Charles Bonnet Syndrome? (Circle all that apply)**

Ophthalmologist

Eye clinic nurse

Optometrist

GP

Social worker

Rehabilitation officer

Sensory awareness team

Friend or family

Macular disease society

Internet research (by self/family/friend)

Radio, television, newspaper or magazine article

Other (please state in the box below)

**17. What type of hallucinations did you / do you see? (Circle all that apply and the duration at its most frequent)**

**Patterns**  once | occasionally | frequently

**Faces**  once | occasionally | frequently

**Shapes**  once | occasionally | frequently

**Figures**  once | occasionally | frequently

**Animals** once | occasionally | frequently

**Objects**  once | occasionally | frequently

Other (please state in the box below)

**18. When did the Charles Bonnet Syndrome start? (years/months)**

(Please state in the box below)

**19. When was your last hallucination? (years/months)**

(Please state in the box below)

**20. Do you consider your hallucinations to have stopped?**

Yes

No

**21. How long did / does each hallucination last on average?**

Seconds

Minutes

Hours

Continuous

**22. When they were/are at their worst, how frequently did/do the typical hallucinations occur? (Circle one that best applies)**

Something was occurring all the time

I had a hallucination almost every hour

I had a hallucination almost every day

I had a hallucination most weeks

I had a hallucination most months

I had a hallucination several times a year or less

**23. What best describes your reaction to Charles Bonnet Syndrome when it first occurred? (Circle all that apply)**

Amused

Curious

Intrigued

Startled

Frightened

Terrified

Indifferent

Frustrated

Other (please state in the box below)

**24. What is your reaction to Charles Bonnet Syndrome now? (Circle all that apply)**

Amused

Curious

Intrigued

Startled

Frightened

Terrified

Indifferent

Frustrated

Other (please state in the box below)

**25. Did / does Charles Bonnet Syndrome interfere with the following abilities? (Circle all that apply)**

Moving about

Watching television

Cooking

Sleeping

Has no effect

Other (please state in the box below)

**26. Charles Bonnet Syndrome had / has the following effect on my life? (Circle the one that best applies)**

A very negative effect

A fairly negative effect

No real effect

A fairly pleasant effect

A very pleasant effect

**27. Who have you told about your hallucinations? (Circle all that apply)**

Medical professionals

Spouse

Other family

Friends

Other people with macular disease

No one

Other (please state in the box below)

**28. What reasons if any have prevented you from telling people? (Circle all that apply)**

Embarrassed by symptoms

Feared others would think I was developing a serious illness e.g. Alzheimer’s or mental problem

I don’t have anyone close to tell

I don’t discuss my health with other people

I was not concerned about it

None

Other (please state in the box below)

**This is the end of Part A, please begin Part B**

**Part B**

**The following is a survey with statements about problems which involve your vision or feelings that you have about your vision.**

**After each question please choose the response that best describes your situation. Please answer all the questions as if you were wearing your glasses or contact lenses (if any).**

**At the present time, do you have:**

**29. Difficulty in reading small print in the telephone book even with glasses?**

No difficulty

A little difficulty

A great difficulty

Unable to perform activity

**30. Difficulty in reading newspaper size print even with glasses?**

No difficulty

A little difficulty

A great difficulty

Unable to perform activity

**31. Difficulty in recognizing friends when you meet them even with glasses?**

No difficulty

A little difficulty

A great difficulty

Unable to perform activity

**32. Difficulty seeing stairs even with glasses?**

No difficulty

A little difficulty

A great difficulty

Unable to perform activity

**33. Difficulty in reading street signs or shop signs even with glasses?**

No difficulty

A little difficulty

A great difficulty

Unable to perform activity

**34. Difficulty in writing cheques or filling out forms even with glasses?**

No difficulty

A little difficulty

A great difficulty

Unable to perform activity

**35. Difficulty in playing games (e.g.chess or cards) even with glasses?**

No difficulty

A little difficulty

A great difficulty

Unable to perform activity

**36. Difficulty in cooking even with glasses?**

No difficulty

A little difficulty

A great difficulty

Unable to perform activity

**37. Difficulty in watching television even with glasses?**

No difficulty

A little difficulty

A great difficulty

Unable to perform activity

**This is the end of Part B, please begin Part C where we will move onto questions about your health and wellbeing OVERALL.**

**Part C**

**This next section is to determine your feelings of your overall wellbeing. Under each heading, please circle the ONE statement that best describes your health TODAY.**

**38. Mobility**

I have no problems in walking about

I have some problems in walking about

I am confined to bed

**39. Self-care**

I have no problems with self-care

I have some problems washing or dressing myself

I am unable to wash or dress myself

**40. Usual activities (e.g. work, study, housework, family or leisure activities)**

I have no problems with performing my usual activities

I have some problems with performing my usual activities

I am unable to perform my usual activities

**41. Pain / discomfort**

I have no pain or discomfort

I have moderate pain or discomfort

I have extreme pain or discomfort

**42. Anxiety / depression**

I am not anxious or depressed

I am moderately anxious or depressed

I am extremely anxious or depressed

**This is the end of Part C, please begin Part D**

**Part D**

**This next set of questions is to determine any feelings of loneliness you may or may not have. After each question please choose the response that best describes your feelings TODAY.**

**43. How often do you feel that you lack companionship?**

Hardly Ever

Some of the Time

Often

**44. How often do you feel left out?**

Hardly Ever

Some of the Time

Often

**45. How often do you feel isolated from others?**

Hardly Ever

Some of the Time

Often

**This is the end of Part D, please begin Part E**

**Part E**

**This section consists of a number of words that describe different feelings and emotions. Read each item and then circle the appropriate answer that describes the way you feel generally, i.e. on average about life.**

*For example:*

**Interested**

Never/Very slightly A little Moderately Quite a bit Extremely

**46. Interested**

Never/Very slightly A little Moderately Quite a bit Extremely

**47. Distressed**

Never/Very slightly A little Moderately Quite a bit Extremely

**48. Excited**

Never/Very slightly A little Moderately Quite a bit Extremely

**49. Upset**

Never/Very slightly A little Moderately Quite a bit Extremely

**50. Strong**

Never/Very slightly A little Moderately Quite a bit Extremely

**51. Guilty**

Never/Very slightly A little Moderately Quite a bit Extremely

**52. Scared**

Never/Very slightly A little Moderately Quite a bit Extremely

**53. Hostile**

Never/Very slightly A little Moderately Quite a bit Extremely

**54. Enthusiastic**

Never/Very slightly A little Moderately Quite a bit Extremely

**55. Proud**

Never/Very slightly A little Moderately Quite a bit Extremely

**56. Irritable**

Never/Very slightly A little Moderately Quite a bit Extremely

**57. Alert**

Never/Very slightly A little Moderately Quite a bit Extremely

**58. Ashamed**

Never/Very slightly A little Moderately Quite a bit Extremely

**59. Inspired**

Never/Very slightly A little Moderately Quite a bit Extremely

**60. Nervous**

Never/Very slightly A little Moderately Quite a bit Extremely

**61. Determined**

Never/Very slightly A little Moderately Quite a bit Extremely

**62. Attentive**

Never/Very slightly A little Moderately Quite a bit Extremely

**63. Jittery**

Never/Very slightly A little Moderately Quite a bit Extremely

**64. Active**

Never/Very slightly A little Moderately Quite a bit Extremely

**65. Afraid**

Never/Very slightly A little Moderately Quite a bit Extremely

**You have finished the Questionnaire.**

**Please check you have filled in all of the questions.**

**Thank you for your time.**
