# Supplementary figures and images for: Emotional well-being in Charles Bonnet syndrome: exploring associations with negative affect, loneliness and quality of life
Source: Ther Adv Ophthalmol. 2024 Sep 26;16:25158414241275444. doi: 10.1177/25158414241275444 (PMC11440537; doi:10.1177/25158414241275444)

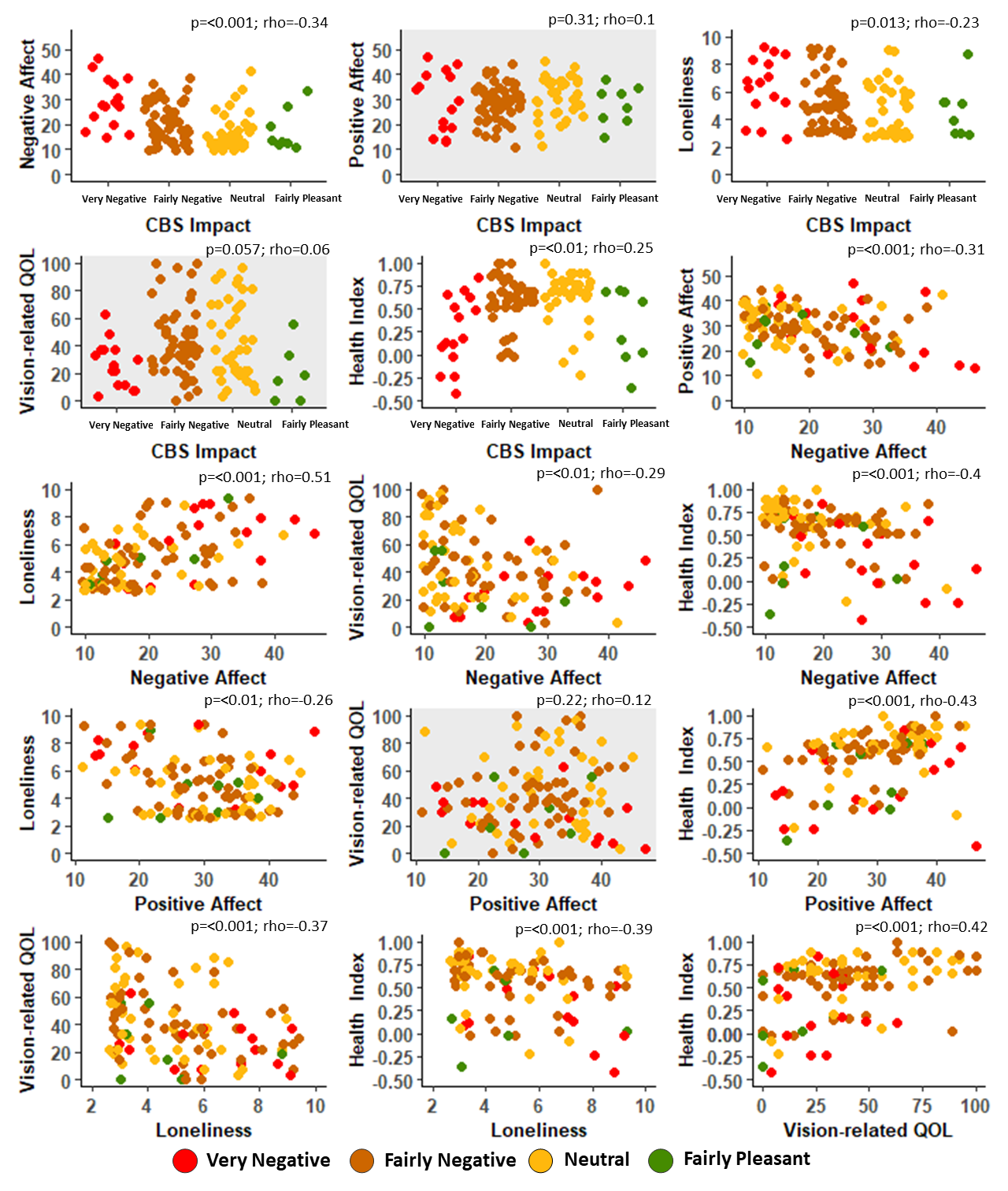

Supplement: sj-tif-2-oed-10.1177_25158414241275444 – Supplemental material for Emotional well-being in Charles Bonnet syndrome: exploring associations with negative affect, loneliness and quality of life [file sj-tif-2-oed-10.1177_25158414241275444.tif]

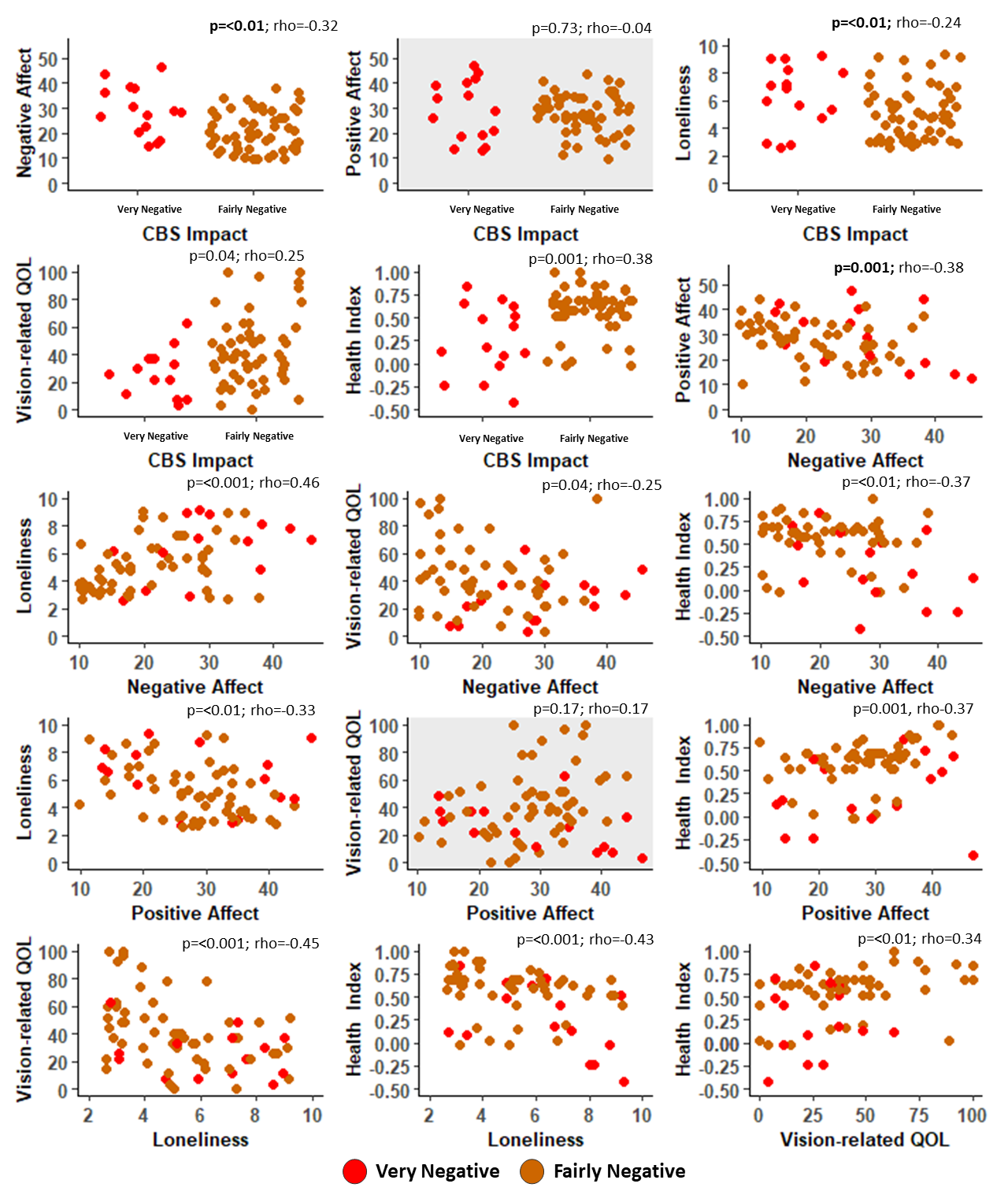

Supplement: sj-tif-3-oed-10.1177_25158414241275444 – Supplemental material for Emotional well-being in Charles Bonnet syndrome: exploring associations with negative affect, loneliness and quality of life [file sj-tif-3-oed-10.1177_25158414241275444.tif]
